# *mcr-1* and *bla*_KPC-3_ in *Escherichia coli* Sequence Type 744 after Meropenem and Colistin Therapy, Portugal

**DOI:** 10.3201/eid2308.170162

**Published:** 2017-08

**Authors:** Marta Tacão, Rafael dos Santos Tavares, Pedro Teixeira, Inês Roxo, Elmano Ramalheira, Sónia Ferreira, Isabel Henriques

**Affiliations:** University of Aveiro, Aveiro, Portugal (M. Tacão, R. dos Santos Tavares, P. Teixeira, E. Ramalheira, I. Henriques);; Centro Hospitalar do Baixo Vouga-EPE, Aveiro (I. Roxo, E. Ramalheira, S. Ferreira);; Instituto de Educação e Cidadania, Aveiro (I. Roxo, S. Ferreira)

**Keywords:** gene, carbapenem, meropenem, colistin, polymixin, fluconazole, linezolid, co-resistance, antimicrobial resistance, antibacterial, antibiotic, IncX4::*mcr-1*, IncF::Tn*1441d***-** *bla*_KPC-3,_ sequence type, Escherichia coli ST744, Klebsiella pneumoniae, multiresistance, Portugal, bacteria

## Abstract

*Escherichia coli* Ec36 was recovered from a patient in Portugal after treatment with meropenem and colistin. Besides an IncF plasmid with Tn1441d-*bla*_KPC-3_, already reported in clinical strains in this country, *E. coli* Ec36 co-harbored an IncX4::*mcr-1* gene. Results highlight emerging co-resistance to carbapenems and polymyxins after therapy with drugs from both classes.

The emergence of the *mcr-1* gene (*1*) and reports on its global dissemination (*2*) unveiled the danger of plasmid-associated colistin resistance. In July 2016, a 70-year-old woman was admitted to the intensive care unit of Centro Hospitalar do Baixo Vouga-EPE, Aveiro, Portugal, for abdominal pain, ostensibly from an abdominal occlusion. After emergency surgery, the patient received meropenem (20 d), fluconazole, and linezolid (both 10 d) and was transferred to the general medicine ward. After 50 days of antibacterial drug therapy, a urine specimen was positive for *Klebsiella pneumoniae* (Kp81). Further testing showed a multidrug-resistance profile, including resistance to carbapenems, but susceptibility to colistin and tigecycline (Table). The drug regimen was altered to colistin and tigecycline for 6 days, after which urine cultures were negative for *K. pneumoniae*. 

**Table:** MIC of antibacterial drugs for *Klebsiella pneumoniae* Kp81, *Escherichia coli* Ec36, transconjugant *E. coli* J53::*mcr-1*, and recipient strain *E. coli* J53 *

Drug	MIC, mg/L (susceptibility)			
*K. pneumoniae *Kp81	*E. coli *Ec 36†	*E. coli *J53::*mcr-1*	*E. coli *J53
Amikacin	≥64 (R)	16 (R)	­ND	ND
Aztreonam	≥64 (R)	≥64 (R)	ND	ND
Cefepime	≥64 (R)	2 (I)	ND	ND
Ceftazidime	≥64 (R)	≥64 (R)	ND	ND
Ciprofloxacin	≥4 (R)	≥4 (R)	ND	ND
Colistin	≤0.5 (S)	8 (R)	4 (R)	0.5 (S)
Gentamicin	≥16 (R)	≥16 (R)	ND	ND
Imipenem	≥16 (R)	≥16 (R)	≤0.25 (S)	≤0.25 (S)
Meropenem	≥16 (R)	≥16 (R)	≤0.25 (S)	≤0.25 (S)
Piperacillin	≥128 (R)	≥128 (R)	ND	ND
Piperacillin/tazobactam	≥128 (R)	≥128 (R)	ND	ND
Ticarcillin	≥128 (R)	≥128 (R)	ND	ND
Ticarcillin/clavulanic acid	≥128 (R)	≥128 (R)	ND	ND
Tigecycline	1.5 (S)	1 (S)	0.25 (S)	0.25 (S)
Tobramycin	≥16 (R)	≥16 (R)	ND	ND
Trimethoprim/sulfamethoxazole	≥320 (R)	≥320 (R)	ND	ND

*MICs were determined by using VITEK2 system AST-N222 (bioMérieux, Marcy-l'Étoile, France), except colistin, for which MICRONAUT MIC-strip (BioConnections, Knypersley, UK) was used, and interpreted according to the European Committee on Antimicrobial Susceptibility Testing (http://www.eucast.org). Shaded rows indicate antibacterial drugs tested for efficacy against each of the 4 organisms. ND, not determined; R, resistant; S, susceptible. †Strain isolated from the patient in this study.

Urine culture was performed as a standard procedure after 72 days. *Escherichia coli* (Ec36) was isolated, showing a resistance profile identical to *K. pneumoniae* Kp81 but expressing colistin resistance (Table). PCR screening and amplicon sequencing confirmed the presence of *mcr-1* in Ec36 and *bla*_KPC-3_ in both isolates (*1*,*3*). All treatments were discontinued, and the patient was discharged 72 days after admission.

We sequenced the Ec36 whole genome (GenBank accession no. MUGF00000000) by using the Illumina HiSeq 2500 platform (Illumina, San Diego, CA, USA); we assembled it de novo by using CLC Genomics (https://www.qiagen.com/us/search/clc-genomics-workbench/) and annotated results by using RAST (http://rast.nmpdr.org/). We used tools available at the Center for Genomic Epidemiology (https://cge.cbs.dtu.dk) to determine the sequence type, resistome, mobilome, serotype, virulence genes, and pathogenicity potential.

Strain Ec36 was assigned to sequence type 744 (ST-744) and predicted as a human pathogen with serotype O89:H10. Testing detected the virulence gene *gad*, encoding a glutamate decarboxylase involved in acid resistance. Besides *mcr-1* and *bla*_KPC-3_, Ec36 harbored genes encoding resistance to aminoglycosides (*strA*, *strB*, *aacA4*, *aadA*, *aadA5*), β-lactams (*bla*_TEM-1B_, *bla*_OXA-9_), macrolides (*mph*[A]), chloramphenicol (*catA1*), tetracycline (*tet*[A], *tet*[B]), sulfonamides (*sul1*, *sul2*), and trimethoprim (*dfrA14*, *dfrA17*). We used Plasmidfinder (https://cge.cbs.dtu.dk/services/PlasmidFinder/) to identify IncX4 (100%; in the *mcr-1*–encoding contig), IncFIA, IncFII, IncQ1, IncX1, and IncI1. We used pMLST 1.4 (https://cge.cbs.dtu.dk/services/pMLST/) to identify IncFIA and IncFII.

*bla*_KPC-3_ was in a 16,455-bp contig, 100% identical to plasmid sequences from clinical *K. pneumoniae* (*4*). In Portugal, this plasmid was reported in clinical isolates of *K. pneumoniae*, *E. coli*, and *Enterobacter* (*5*). *bla*_KPC-3_ was part of Tn*4401* isoform d (*4*), flanked by IS*Kpn7* and IS*Kpn6* and located in a cointegrated FIA and FII plasmid (pEc36-KPC3), co-harboring *bla*_TEM_, *bla*_OXA-9_, *aacA4*, and *aadA1*. We analyzed the genetic context of *bla*_KPC-3_ in Kp81 and Ec36 by using a PCR-based protocol (*4*), which indicated a similar context in both strains within Tn*4401d* in a FIA-FII plasmid. As highlighted previously (*5*), results reinforce the role of Tn*4401d* on the spread of carbapenemase genes among *Enterobacteriaceae* in Portugal.

We identified the *mcr-1* gene in a 9,085-bp contig, which matched *E. coli* SHP45 100% (*1*). Genetic context analysis identified a 2,600-bp *mcr-1*–containing cassette recognized in different plasmid backbones (*6*), suggesting its mobilization between different hosts.

The IncX4 plasmid harboring *mcr-1* (pEc36_mcr-1) was divided into 2 contigs, which we subsequently cloned by using PCR and sequencing. pEc36_mcr-1 was 33,140-bp and had no other resistance genes, nor IS*Ap11*, found originally associated with *mcr*-1 and linked to animal reservoirs (*7*). Plasmid sequence showed high similarity to pESTMCR (GenBank accession no. KU743383), pMCR1-IncX4 (accession no. KU761327), and pMCR1-NJ-IncX4 (accession no. KX447768).

We performed mating assays by using Ec36 as donor and *E. coli* J53 as recipient. Transconjugants were obtained in Plate-Count-Agar (Merck, Germany) with sodium azide (100 mg/L) and colistin (2 mg/L). The MIC of colistin for the transconjugant (4 mg/L) was 8 times higher than that for *E. coli* J53. We detected *mcr-1* by using PCR for the transconjugant, but not *bla*_KPC-3_.

*mcr-1* was previously detected in carbapenem-susceptible *E. coli* ST744 in Denmark (*8*) and in *E. coli* ST744, co-producing CTX-M–like β-lactamases, in Taiwan (*9*). Regarding clinical *mcr*-1–positive *E*. *coli*, >10 STs have been reported, including the high-risk ST-131 (*8*,*9*). Therefore, the association of a successful clone to the spread of *mcr-1* is not evident, but apparently, it is associated with successful plasmids (e.g., IncX4).

In Portugal, *mcr-1* has been reported in *Salmonella* and *E. coli* from food products and in clinical *Salmonella* isolates (*2*,*10*). Since *bla*_KPC-3_ is increasingly reported in Portugal, its co-occurrence with *mcr-1*–harboring plasmids represents a serious concern.

*mcr-1* has been found in isolates that produce carbapenemases KPC, NDM, VIM, and OXA-48 (*2*,*7*). Carbapenemase genes usually are associated with mobile elements that encode resistance to several antibacterial drugs, and consequently produce multiresistance traits, as in *E. coli* Ec36. This scenario might predict the emergence of drug-resistant phenotypes, likely jeopardizing treatment.

In summary, we isolated KPC-3–producing and *mcr*-*1*–harboring *E. coli* Ec36 from a patient after treatment with meropenem, then colistin. Colistin-resistant Ec36 may have been part of the patient’s gut microbiome, acquiring the *bla*_KPC-3_-encoding plasmid from the KP81 strain. Although neutropenic, the patient’s samples showed an asymptomatic bacteriuria. Thus, prophylactic administration of antibacterial drugs was likely avoidable.
